# Clustering of geriatric deficits emerges to be an essential feature of ageing - results of a cross-sectional study in Poland

**DOI:** 10.18632/aging.101055

**Published:** 2016-10-27

**Authors:** Piotrowicz Karolina, Pac Agnieszka, Skalska Anna Barbara, Chudek Jerzy, Klich-Rączka Alicja, Szybalska Aleksandra, Michel Jean-Pierre, Grodzicki Tomasz

**Affiliations:** ^1^ Department of Internal Medicine and Gerontology, Jagiellonian University Medical College, 31-531 Krakow, Poland; ^2^ Department of Epidemiology and Preventive Medicine, Jagiellonian University Medical College, 31-034 Kraków, Poland; ^3^ Department of Pathophysiology, Medical Faculty in Katowice, Medical University of Silesia in Katowice, 40-752 Katowice, Poland; ^4^ Department of Nephrology, Endocrinology and Metabolic Diseases, Medical Faculty in Katowice, Medical University of Silesia in Katowice, 40-027 Katowice, Poland; ^5^ International Institute of Molecular and Cell Biology, 02-109 Warsaw, Poland; ^6^ Geneva Medical School and University Hospitals - Rehabilitation and Geriatrics, Geneva, Switzerland

**Keywords:** geriatric deficit, geriatric impairment, geriatric syndrome, epidemiology, deficit accumulation

## Abstract

The majority of old people suffer from various clinical conditions that affect health, functioning and quality of life. This research is a part of a cross-sectional, nationwide PolSenior Study that provides a comprehensive assessment of eight geriatric impairments and their co-occurrence in a representative sample (3471 participant aged 65-104 years, mean age 78.3 years) of the old adults living in the community in Poland. The participants were recruited randomly from all administrative regions of Poland by a three-stage, proportional, stratified-by-age group selection process. Eight geriatric conditions were assessed: falls, incontinences, cognitive impairment, mood disorders, vision and hearing impairments, malnutrition, and functional dependence. We showed that the most common deficits causing disability were vision and hearing impairments, and mood disorders, with more than two thirds of the participants presented at least one geriatric deficit. We showed that presence any of the analyzed conditions significantly increased the risk for co-occurrence of other examined weaknesses. The highest prevalence odds ratios were for functional dependence and, respectively: malnutrition (8.61, 95%CI: 4.70-15.80), incontinences (8.0, 95%CI:5.93-10.70), and cognitive impairment (7.22; 95%CI:5.91-8.83). We concluded that the majority of the old people living in the community present various clinical conditions that prompt disability.

## INTRODUCTION

Cognitive and functional status is getting worse with age, hence the number of existing impairments leading to the geriatric syndromes such as delirium, falls, incontinence, functional decline, or pressure ulcers is rising as well [1]. From the epidemiological point of view, the number of coexistent diseases seems the most important, whereas from individual perspective, not only the number, but also the nature and a specific combination of deficits present in old adults is crucial to create the systems to provide the best care [2,3].

As noted by Chatterji and colleagues we urgently need studies evaluating of either compression or expansion of morbidity in the old adults as data assessing cumulative frequency of geriatric disorders that require comprehensive assessment, and consequently, interdisciplinary care is limited [4].

Taking into account the complex and interactive causes of disability manifesting in old age, the authors selected some important geriatric conditions and outlined an overview of the issue of age-related impairments in the community-dwelling old adults living in Poland.

The aim of the analysis was to assess the prevalence and coexistence of the geriatric conditions, showed as the frequency of co-occurrence of impairments in the population of Polish older people aged 65 years and more.

## RESULTS

The study sample consisted of 3471 respondents, 52.3% of them were men. Mean age was 78.3±8.4 years (min-max: 65-104). Mean age according to sex was 78±8.5 years in women and 78.5±8.3 years in men.

The prevalence of all the described clinical conditions rose along with the increase of age. The most common was: vision impairment, even if wearing glasses (almost 42% of all analyzed subjects suffered from various degrees of vision deterioration), then mood disorders (27%), hearing impairment (22%), and cognitive impairment (21%). Malnutrition, based on low albumin concentration, was relatively rare among respondents examined.

The constellation of the deficits varied in particular age groups. In the group of 65-79-year-old respondents, the most frequent were the following: vision deficits, mood disorders, and auditory impairments. In those aged 80-89, they were respectively: sensory deficits (both hearing and vision problems, with the latter affecting almost half of the population) and, with similar frequency, functional dependence, cognitive impairment, and mood disorders. Among the oldest olds the most frequent problem was limitation in functional independence, then hearing deficits, cognitive impairment, and vision impairments (Table 1).

**Table 1:** The prevalence (%, 95%CI) of analyzed geriatric conditions in the whole group and in age subgroups (weighted data)

	All (n=3471)	65-79 years (n=1962)	80-89 years (n=1094)	90+ years (n=415)
**Vision impairment (%)**	41.6 (39.8 to 43.4)	39.2 (37.0 to 41.4)	48.0 (44.6 to 51.4)	57.1 (51.8 to 62.2)
**Mood disorders (%)**	27.2 (25.6 to 28.8)	25.5 (23.6 to 27.5)	31.5 (28.5 to 34.6)	37.4 (32.5 to 42.6)
**Hearing impairment (%)**	21.9 (20.5 to 23.4)	16.3 (14.7 to 18.0)	36.2 (33.1 to 39.5)	60.1 (54.9 to 65.1)
**Cognitive impairment (%)**	20.7 (19.3 to 22.1)	15.9 (14.3 to 17.6)	32.0 (29.0 to 35.2)	58.4 (53.2 to 63.5)
**Falls (%)**	17.3 (16.0 to 18.6)	14.1 (12.6 to 15.7)	26.2 (23.3 to 29.2)	35.2 (30.4 to 40.3)
**Functional dependence (%)**	17.1 (15.8 to 18.3)	10.7 (9.4 to 12.1)	32.8 (29.8 to 35.9)	64.5 (59.3 to 69.4)
**Incontinences (%)**	5.9 (5.1 to 6.6)	3.7 (3.0 to 4.7)	11.0 (9.2 to 13.1)	22.2 (18.3 to 26.6)
**Malnutrition (%)**	1.5 (1.1 to 1.9)	1.1 (0.7 to 1.7)	2.3 (1.4 to 3.5)	6.8 (4.6 to 9.9)
**Number of impairments**	1.36 (1.32 to 1.40)	1.16 (1.11 to 1.21)	1.87 (1.78 to 1.96)	2.77 (2.61 to 2.93)

**Figure 1:**
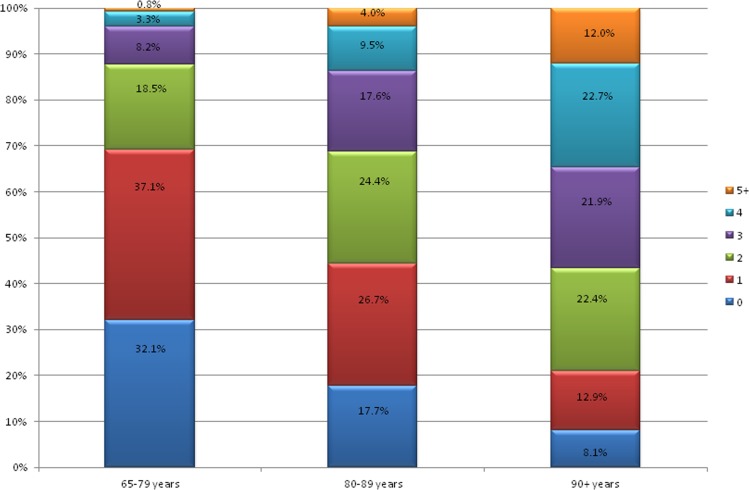
The percentage of subjects with the listed total number of analyzed geriatric conditions (from 0 to 5 and more) in age subgroups: 65-79 years, 80-89 years and 90+ years old

In more than two-thirds of the examined senior respondents at least one geriatric problem was revealed; while, more than one-third manifested two or more geriatric deficits; none of respondents had all eight impairments. On the other hand, 32% of those aged 65-79 years, almost 18% of those aged 80-89 years and 8% of the oldest olds had no geriatric condition assessed in this study. While among those younger than 90 years of age the majority of studied respondents presented one geriatric disability, in those aged 90 years and more, those with two and four geriatric problems prevailed (Figure 1).

The cumulative number of geriatric impairments rose with age, and was respectively: 1.16, 1.87 and 2.77 in the age subgroups (Table 1).

In logistic regression models, all conditions significantly increased the risk of other geriatric problem co-occurrence. Vision impairment significantly increased the risk for co-occurrence of all, except for incontinences and malnutrition, while cognitive impairment posing the greatest risk here (OR-2.07).

Mood disorders, hearing impairment, and cognitive impairment enhanced the risk for all the analyzed conditions. The three above conditions highly increased the risk of functional dependence (OR respectively: 2.69, 3.82 and 7.22).

Falls and functional dependence increased the risk for all analyzed conditions; the strongest relation was between falls and incontinences (OR-3.72), and functional dependence and malnutrition (OR-8.61) respectively.

Both incontinences and malnutrition raised the risk for all except vision impairment, with the greatest risk of co-occurrence between them and functional dependence (OR respectively: 8.0 and 8.61). The results were presented in Table 2.

**Table 2:** The conditional probabilities (%, 95%CI) and prevalence odds ratios (95%CI) for analyzed geriatric conditions. (The percentages represent the frequency of cases described in the columns among those with the conditions given in the rows.)

		Vision impairment	Mood disorders	Hearing impairment	Cognitive impairment	Falls	Functional dependence	Incontinences	Malnutrition
**Vision impairment**	Y (%)	NA	33.7 (31.1 to 36.4)	25.7 (23.4 to 28.1)	27.7 (25.4 to 30.2)	19.2 (17.1 to 21.3)	23.1 (21.0 to 25.3)	6.4 (5.3 to 7.7)	1.6 (1.0 to 2.4)
	N (%)		22.5 (20.6 to 24.6)	19.3 (17.5 to 21.2)	15.6 (14.0 to 17.4)	16.0 (14.4 to 17.8)	12.8 (11.4 to 14.3)	5.5 (4.6 to 6·6)	1.5 (1.0 to 2.2)
	OR		**1.75 (1.48 to 2.06)**	**1.45 (1.22 to 1.73)**	**2.07 (1.73 to 2.47)**	**1.24 (1.03 to 1.50)**	**2.05 (1.70 to 2.47)**	**1.18 (0.89 to 1.50)**	**1.05 (0.60 to 1.86)**
**Mood disorders**	Y (%)	51.6 (48.2 to 55.1)	NA	27.5 (24.6 to 30.5)	31.2 (28.2 to 34.3)	27.2 (24.4 to 30.3)	28.4 (25.5 to 31.4)	8.2 (6.6 to 10.2)	2.5 (1.7 to 3.8)
	N (%)	37.9 (35.8 to 40.0)		19.9 (18.3 to 21.6)	16.8 (15.3 to 18.4)	13.6 (12.3 to 15.1)	12.8 (11.6 to 14.2)	5.0 (4.2 to 5.9)	1.2 (0.8 to 1.7)
	OR	**1.75 (1.48 to 2.06)**		**1.53 (1.27 to 1.84)**	**2.25 (1.87 to 2.70)**	**2.37 (1.96 to 2.88)**	**2.69 (2.22 to 3.25)**	**1.72 (1.28 to 2.30)**	**2.19 (1.25 to 3.86)**
**Hearing impairment**	Y (%)	48.8 (45.0 to 52.5)	34.0 (30.6 to 37.6)	NA	36.5 (33.1 to 40.1)	23.7 (20.8 to 26.8)	34.5 (31.3 to 38.0)	12.7 (10.6 to 15.0)	3.3 (2.3 to 4.8)
	N (%)	39.6 (37.6 to 41.7)	25.2 (23.5 to 27.1)		16.2 (14.8 to 17.8)	15.5 (14.1 to 17.1)	12.1 (10.9 to 13.5)	3.9 (3.3 to 4.8)	1.0 (0.7 to 1.5)
	OR	**1.45 (1.22 to 1.73)**	**1.53 (1.27 to 1.84)**		**2.97 (2.46 to 3.59)**	**1.69 (1.38 to 2.06)**	**3.82 (3.15 to 4.63)**	**3.53 (2.66 to 4.60)**	**3.34 (1.90 to 5.86)**
**Cognitive impairment**	Y (%)	55.8 (52.0 to 59.6)	40.9 (37.2 to 44.7)	38.8 (35.2 to 42.5)	NA	26.5 (23.4 to 29.9)	44.3 (40.7 to 48.1)	14.1 (11.8 to 16.7)	2.5 (1.7 to 3.7)
	N (%)	37.9 (35.9 to 40.0)	23.6 (21.9 to 25.4)	17.6 (16.1 to 19.1)		14.9 (13.5 to 16.4)	9.9 (8.8 to 11.2)	3.7 (3.1 to 4.5)	1.3 (0.9 to 1.8)
	OR	**2.07 (1.73 to 2.47)**	**2.25 (1.87 to 2.70)**	**2.97 (2.46 to 3.59)**		**2.06 (1.68 to 2.52)**	**7.22 (5.91 to 8.83)**	**4.27 (3.21 to 5.60)**	**1.99 (1.15 to 3.45)**
**Falls**	Y (%)	46.1 (42.0 to 50.2)	42.7 (38.7 to 46.9)	30.0 (26.5 to 33.8)	31.7 (28.0 to 35.5)	NA	34.7 (31.0 to 38.6)	13.9 (11.4 to 16.9)	3.6 (2.4 to 5.5)
	N (%)	40.7 (38.7 to 42.7)	23.9 (22.2 to 25.7)	20.3 (18.7 to 21.9)	18.4 (16.9 to 19.9)		13.4 (12.2 to 14.7)	4.17 (3.5 to 5.0)	1.1 (0.7 to 1.6)
	OR	**1.24 (1.03 to 1.50)**	**2.37 (1.96 to 2.88)**	**1.69 (1.38 to 2.06)**	**2.06 (1.68 to 2.52)**		**3.45 (2.81 to 4.23)**	**3.72 (2.77 to 4.90)**	**3.45 (1.95 to 6.10)**
**Functional dependence**	Y (%)	56.3 (52.3 to 60.3)	45.2 (41.2 to 49.2)	44.4 (40.5 to 48.4)	53.8 (49.7 to 57.8)	35.2 (31.5 to 39.2)	NA	19.8 (16.9 to 23.0)	5.6 (4.0 to 7.7)
	N (%)	38.6 (36.6 to 40.6)	23.5 (21.8 to 25.2)	17.3 (15.8 to 18.9)	13.9 (12.5 to 15.3)	13.6 (12.3 to 15.0)		3.0 (2.4 to 3.7)	0.7 (0.4 to 1.1)
	OR	**2.05 (1.70 to 2.47)**	**2.69 (2.22 to 3.25)**	**3.82 (3.15 to 4.63)**	**7.22 (5.91 to 8.83)**	**3.45 (2.81 to 4.23)**		**8.0 (5.93 to 10.70)**	**8.61 (4.70 to 15.80)**
**Incontinences**	Y (%)	45.4 (38.8 to 52.1)	38.2 (31.8 to 45.0)	47.4 (40.8 to 54.1)	49.8 (43.1 to 56.5)	38.0 (29.0 to 47.9)	57.6 (50.7 to 64.3)	NA	7.2 (4.6 to 11.1)
	N (%)	41.4 (39.5 to 43.3)	26.5 (24.9 to 28.2)	20.4 (18.9 to 21.9)	18.9 (17.5 to 20.3)	13.8 (12.2 to 15.5)	14.5 (13.3 to 15.8)		1.2 (0.8 to 1.6)
	OR	**1.18 (0.89 to 1.50)**	**1.72 (1.28 to 2.30)**	**3.53 (2.66 to 4.60)**	**4.27 (3.21 to 5.60)**	**3.72 (2.77 to 4.90)**	**8.0 (5.93 to 10.70)**		**6.52 (3.61 to 11.70)**
**Malnutrition**	Y (%)	42.9 (30.0 to 56.8)	33.9 (22.9 to 46.8)	47.8 (34.4 to 61.5)	44.6 (31.5 to 58.6)	41.3 (28.7 to 55.2)	62.7 (48.0 to 75.4)	27.6 (17.7 to 40.3)	NA
	N (%)	41.6 (39.8 to 43.5)	20.5 (19.1 to 21.9)	21.5 (20.1 to 23.0)	26.9 (25.3 to 28.5)	16.9 (15.7 to 18.3)	16.3 (15.2 to 17.6)	5.5 (4.8 to 6.3)	
	OR	**1.05 (0.60 to 1.86)**	**2.19 (1.25 to 3.86)**	**3.34 (1.90 to 5.86)**	**1.99 (1.15 to 3.45)**	**3.45 (1.95 to 6.10)**	**8.61 (4.70 to 15.80)**	**6.52 (3.61 to 11.70)**	

Y-yes, N-no, OR- odd ratio, NA- not applicable.

All prevalence odds ratios for the co-occurrence of geriatric conditions in the whole group and for age subgroups were presented in Table 2 and Table 3.

**Table 3:** The prevalence odds ratios (95%CI) for analyzed geriatric conditions in age cohorts: 65-79 years (n=1962), 80-89 years (n=1094) and 90+ years old (n=415)

		Vision impairment	Mood disorders	Hearing impairment	Cognitive impairment	Falls	Functional dependence	Incontinences	Malnutrition
**Vision impairment**	65-79	NA	1.75 (1.42 to 2.15)	1.34 (1.04 to 1.71)	1.81 (1.41 to 2.32)	1.07 (0.83 to 1.39)	2.02 (1.50 to 2.71)	1.13 (0.70 to 1.80)	0.79 (0.31 to 2.00)
	80-89		1.58 (1.19 to 2.11)	1.26 (0.95 to 1.66)	2.27 (1.70 to 3.03)	1.41 (1.04 to 1.90)	1.70 (1.28 to 2.25)	0.98 (0.65 to 1.40)	1.50 (0.60 to 3.77)
	90+		1.83 (1.17 to 2.88)	1.51 (0.98 to 2.32)	1.73 (1.12 to 2.67)	1.00 (0.65 to 1.56)	1.63 (1.04 to 2.55)	0.75 (0.46 to 1.20)	0.58 (0.25 to 1.32)
**Mood disorders**	65-79	1.75 (1.42 to 2.15)	NA	1.40 (1.08 to 1.83)	2.46 (1.90 to 3.17)	2.50 (1.92 to 3.25)	3.22 (2.39 to 4.33)	1.75 (1.08 to 2.80)	1.95 (0.79 to 4.77)
	80-89	1.58 (1.19 to 2.11)		1.48 (1.10 to 1.98)	1.68 (1.25 to 2.26)	1.97 (1.44 to 2.70)	1.92 (1.43 to 2.57)	1.59 (1.05 to 2.40)	2.05 (0.83 to 5.07)
	90+	1.83 (1.17 to 2.88)		1.43 (0.92 to 2.23)	1.75 (1.11 to 2.75)	1.70 (1.09 to 2.67)	2.61 (1.60 to 4.24)	0.83 (0.50 to 1.30)	2.20 (0.98 to 4.95)
**Hearingimpairment**	65-79	1.34 (1.04 to 1.71)	1.40 (1.08 to 1.83)	NA	2.60 (1.95 to 3.46)	1.30 (0.94 to 1.80)	2.93 (2.12 to 4.06)	2.37 (1.41 to 3.90)	2.76 (1.09 to 6.98)
	80-89	1.26 (0.95 to 1.66)	1.48 (1.10 to 1.98)		1.90 (1.42 to 2.55)	1.40 (1.02 to 1.90)	2.40 (1.80 to 3.20)	2.71 (1.79 to 4.00)	2.66 (1.04 to 6.78)
	90+	1.51 (0.98 to 2.32)	1.43 (0.92 to 2.23)		2.84 (1.82 to 4.44)	1.81 (1.14 to 2.86)	3.29 (2.07 to 5.22)	3.37 (1.92 to 5.90)	1.75 (0.70 to 4.35)
**Cognitive impairment**	65-79	1.81 (1.41 to 2.32)	2.46 (1.90 to 3.17)	2.60 (1.95 to 3.46)	NA	1.88 (1.38 to 2.56)	6.72 (4.92 to 9.18)	3.37 (2.05 to 5.50)	1.22 (0.40 to 3.72)
	80-89	2.27 (1.70 to 3.03)	1.68 (1.25 to 2.26)	1.90 (1.42 to 2.55)		1.49 (1.09 to 2.04)	4.62 (3.41 to 6.26)	3.19 (2.11 to 4.80)	1.13 (0.46 to 2.78)
	90+	1.73 (1.12 to 2.67)	1.75 (1.11 to 2.75)	2.84 (1.82 to 4.44)		1.86 (1.18 to 2.93)	5.30 (3.27 to 8.60)	2.97 (1.68 to 5.20)	6.22 (1.90 to 20.34)
**Falls**	65-79	1.07 (0.83 to 1.39)	2.50 (1.92 to 3.25)	1.30 (0.94 to 1.80)	1.88 (1.38 to 2.56)	NA	3.65 (2.64 to 5.05)	4.39 (2.70 to 7.10)	3.35 (1.37 to 8.22)
	80-89	1.41 (1.04 to 1.90)	1.97 (1.44 to 2.70)	1.40 (1.02 to 1.90)	1.49 (1.09 to 2.04)		2.09 (1.53 to 2.85)	2.32 (1.53 to 3.50)	3.44 (1.40 to 8.47)
	90+	1.00 (0.65 to 1.56)	1.70 (1.09 to 2.67)	1.81 (1.14 to 2.86)	1.86 (1.18 to 2.93)		2.26 (1.39 to 3.69)	1.48 (0.90 to 2.40)	1.11 (0.47 to 2.58)
**Functional dependence**	65-79	2.02 (1.50 to 2.71)	3.22 (2.39 to 4.33)	2.93 (2.12 to 4.06)	6.72 (4.92 to 9.18)	3.65 (2.64 to 5.05)	NA	5.71 (3.47 to 9.40)	10.03 (4.16 to 24.19)
	80-89	1.70 (1.28 to 2.25)	1.92 (1.43 to 2.57)	2.40 (1.80 to 3.20)	4.62 (3.41 to 6.26)	2.09 (1.53 to 2.85)		6.12 (3.90 to 9.50)	3.85 (1.42 to 10.43)
	90+	1.63 (1.04 to 2.55)	2.61 (1.60 to 4.24)	3.29 (2.07 to 5.22)	5.30 (3.27 to 8.60)	2.26 (1.39 to 3.69)		13.30 (5.17 to 34.20)	11.46 (1.52 to 86.61)
**Incontinences**	65-79	1.13 (0.70 to 1.80)	1.75 (1.08 to 2.80)	2.37 (1.41 to 3.90)	3.37 (2.05 to 5.50)	4.39 (2.70 to 7.10)	5.71 (3.47 to 9.40)	NA	3.92 (1.11 to 13.80)
	80-89	0.98 (0.65 to 1.40)	1.59 (1.05 to 2.40)	2.71 (1.79 to 4.00)	3.19 (2.11 to 4.80)	2.32 (1.53 to 3.50)	6.12 (3.90 to 9.50)		7.50 (3.01 to 18.6)
	90+	0.75 (0.46 to 1.20)	0.83 (0.50 to 1.30)	3.37 (1.92 to 5.90)	2.97 (1.68 to 5.20)	1.48 (0.90 to 2.40)	13.30 (5.17 to 34.20)		2.96 (1.29 to 6.70)
**Malnutrition**	65-79	0.79 (0.31 to 2.00)	1.95 (0.79 to 4.77)	2.76 (1.09 to 6.98)	1.22 (0.40 to 3.72)	3.35 (1.37 to 8.22)	10.03 (4.16 to 24.19)	3.92 (1.11 to 13.80)	NA
	80-89	1.50 (0.60 to 3.77)	2.05 (0.83 to 5.07)	2.66 (1.04 to 6.78)	1.13 (0.46 to 2.78)	3.44 (1.40 to 8.47)	3.85 (1.42 to 10.43)	7.50 (3.01 to 18.6)	

NA- not applicable.

## DISCUSSION

Our survey showed that more than two thirds of seniors living in the community suffered from at least one geriatric problem, noteworthy more than one third had two or more deficits.

The coexistence of the impairments was particularly prevalent in those in the tenth decade of life of whom less than 10% remained in good health and mental condition.

The main reasons for loss of independence were vision and hearing deficits, and mood disorders. The prevalence and also cumulative number of the examined impairments rose with age, with the latter accounting for almost three in the oldest olds.

In the regression logistic models, we showed that respondents who presented any of the tested deficits had significantly higher risk of co-occurrence of other listed geriatric troubles. Along with the diagnosis of any of the analyzed geriatric problems rose the conditional probabilities and odds ratios for other described deficits. The greatest risk was observed for functional dependence, being both a consequence and predisposing factor of geriatric symptoms.

To the best of our knowledge, there is a lack of research estimating the reciprocal conditional probabilities and prevalence odds ratios for multiple geriatric conditions in a representative sample of respondents living in the community.

We searched for reports published in English, relating to the concurrence of the geriatric impairments in the community-dwelling respondents, with the keywords: geriatric problems, geriatric giants, geriatric deficits, geriatric syndromes, geriatric conditions, geriatric disabilities, geriatric impairments.

Our results might be compared to the results of the Health and Retirement Study (HRS). In administered in 2000 the HRS survey older Americans living in the community and in nursing homes were checked, in the context of Activities of Daily Living dependency, for geriatric conditions such as cognitive impairment, falls, incontinence, low body mass index, dizziness, and sensory impairments. The authors showed that. almost 50% of the examined older Americans had at least one geriatric condition - 30% of respondents presented one, 12% showed two and 7% three and more geriatric problems [5]. When compared the prevalence trends of the diseases and impairments in the three different waves of the HRS study (1998, 2004 and 2008), the decrease in the prevalence rate of those free of impairments was shown (from 47.3% in 1998, 45.3% in 2004 to 44.4% in 2008) [6].

The higher prevalence and incidence of eight geriatric conditions (i.e. cognitive impairment, falls, incontinence, low body mass index, dizziness, vision and hearing impairment, pain) were found in the middle-aged and older-aged diabetic participants of 2004 wave of the HRS study when compared to those without diabetes [7].

In the Women's Health Initiative Observational Study (WHI-OS) conditional probabilities for co-occurrence of ten geriatric problems were tested (i.e. depressive symptoms, dizziness, falls, hearing or visual impairment, osteoporosis, polypharmacy, syncope, sleep disturbance, urinary incontinence). Nevertheless, the results were not representative for the general population of old people living in the community, as the studied group comprised of women who were free of any limitation in the Activities of Daily Living scale [8].

PolSenior Project was the nationwide epidemiological study of Polish old people living in the community, providing essential information to both physicians and health care policy makers on the health, social and economic situation of senior citizens [9]. Information concerning the prevalence and subsequent risk of co-occurrence of the described clinical conditions seems crucial from the medical and socio-economic points of view.

Realizing the fact that most of the community-dwelling older adults suffer from some geriatric troubles, should motivate the health care providers to start up with the widespread screening and preventive strategies.

Understanding and identifying symptom and impairment clusters, will help us to predict occurrence of co-existing syndromes and geriatric scenarios (defined and called by the authors ‘*obstacles*’) that are most likely to happen. Both ageing and aged patients should be perceived not as those with two or more geriatric problems that require the separate treatment strategies, but as subjects with complex cumulative deficits who require individualized, patient-centered care [10–12].

When discussing the conditions assessed in our study, it was visible that all of them can be treated or diminished to some extent. Admittedly, modern medicine is able only to slower or moderate the course of dementia and dementia related behaviors, but it is manageable to correct vision almost completely and improve hearing loss. It is also possible to reduce the risk and treat malnutrition or provide some rehabilitation programs targeted at falls and frailty prevention [13–17]. Considering the frequency of geriatric syndromes and their inevitably unfavorable clinical course, the importance of effective preventive strategies should be emphasized. Despite the multifactorial pathogenesis of geriatric impairments, the current knowledge of its mechanisms favors prevention and treatment of vascular ageing and vascular disease (i.e. adequate blood pressure control, statins, antiplatelet drugs, healthy lifestyle with balanced diet and physical activity), being the common pathway in many geriatric scenarios [18]. Still, more research is needed on the multicomponent preventive and treatment strategies to maintain healthy ageing and prevent disability in old age, the Finnish Geriatric Intervention Study to Prevent Cognitive Impairment and Disability (FINGER) and the Sarcopenia and Physical fRailty IN older people: multi-componenT Treatment strategies (SPRINTT) studies being relevant [19,20].

Due to the worse prognosis of older persons burdened with the geriatric syndromes economic aspect is also important. There are some economic evaluations available pointing out that in the US the societal costs of treatment and care for old demented people are similar to or even exceed the financial burden related to cancers or heart disease [21]. It seems extremely important, especially in the light of data revealing that in Poland hospital care for senior citizens, who constituted merely 13% of the society, devoured 34% of the health care costs, making the last phase of life very costly [22]. From cost-efficiency perspective, preventing or shortening hospitalization, decreasing the rate of re-hospitalization, reducing demand for post-hospitalization help due to better geriatric care and geriatrics-related services may significantly reduce expenditures on seniors [23–25]. Also the aspect of senior citizens' and their caregivers' quality of life should not be neglected [26].

There were some limitations to this study.

As it was a cross-sectional, multicenter, community-based study, we performed the routine screening examination of cognition and mood, vision and hearing, and functional dependence. There are no results of further detailed assessment available.

Secondly, the estimates of the prevalence of impairments in Polish older people might be lowered as the patients with diagnosis of severe cognitive impairment or even the end stage dementia, in whom some deficits were impossible to determine, were included in analyses (classified as “ineligible”). We decided not to exclude those respondents from our study in order to preserve the structure of the group representative for the general population of Polish old adults.

Additionally, the number of assessed deficits could be also underestimated as there was missing data due to some problems with blood collection from severely disabled or demented respondents during home visits. The excluded respondents were older [the oldest olds were excluded more often (46.0%) than those aged 65-79 years (23.0%) and those 80-89 years old (34.2%)] and generally were in poorer health condition.

Our results demonstrated the fact of accumulation of deficits in the old adults, with reciprocal conditional probabilities between them. The proportion of respondents burdened with the geriatric problems rose with age, reaching 90% in among the oldest old.

## MATERIALS AND METHODS

### Study design, participants

The PolSenior Project was the first nationwide, multicenter cross-sectional, community-dwelling research carried out between 2007 and 2011, by 40 research groups, that evaluated medical, psychological and socioeconomic aspects of ageing in Poland.

The study was conducted on 5695 subjects participating in the PolSenior Project, who had been randomly selected from 16 administrative regions of Poland by three-stage, proportional, and stratified –by age group selection process, as described elsewhere [9]. In brief, stratified random sampling was used with the aim of recruiting old men and women and putting them into six age-groups spanning five years each: 65–69, 70–74, 75–79, 80–84, 85–89, and 90 years and over, and also those of 55-59 years of age, as a comparative sample. The youngest cohort (55-59 years old, 716 respondents) was not included in the present analysis. Old adults (4797 probates) were answering an interviewer-administered questionnaire, had blood and urine sampling taken whenever feasible. All procedures were performed by pre-trained nurses.

The study fully complied with all applicable institutional and governmental regulations concerning the ethical use of human volunteers and with the terms of the Helsinki Declaration. The institutional review board approved the study protocol (the Bioethics Committee of the Medical University of Silesia in Katowice, Poland; number: KNW-6501-38/I/08) and all the recruited subjects gave their written informed consent.

**Figure 2:**
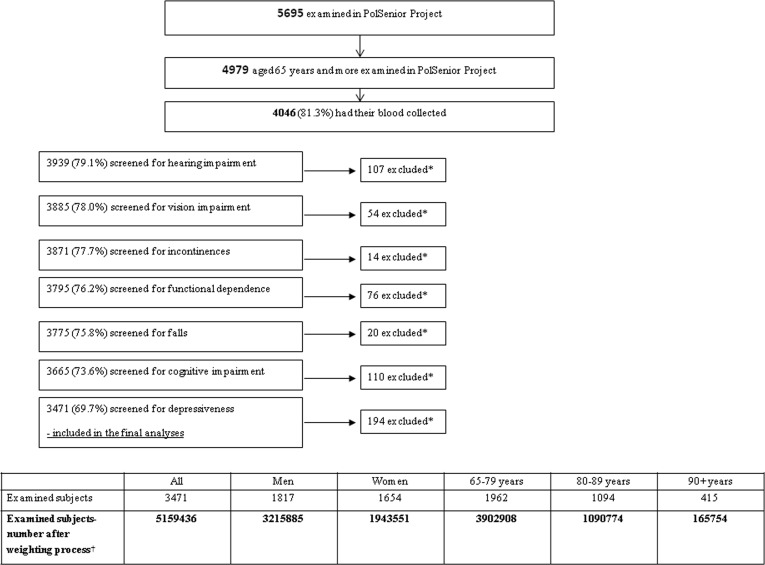
Group selection process * Exclusion due to lack of complete data. † Weights for gender and age have been applied to produce nationally representative estimates for population aged 65 years and older.

Respondents with missing data in one of eight variables used in this analysis were excluded from this study. Finally, from 4046 subjects in whom albumin concentration was measured, 3471 were included in the analysis. Detailed selection process was presented in Figure 2. There was no significant difference according to sex when comparing those included and excluded from the analyses.

For the purpose of this analysis, the included respondents were subsequently divided into three age subgroups: 65-79 year-olds (1962 subjects), 80-89 year-olds (1094 probates), and 90 year-olds and more (415 subjects).

### Variables, methods of assessment

#### The idea of obstacle acronym

The presented analysis concerns the prevalence of eight clinical conditions observed among old adults the most frequently.

From the impairments selected for the study, we composed the acronym OBSTACLE, which may stand for the most common difficulties faced in the every-day life by senior citizens.

Deficits examined in the study included: O like ocular disease (vision impairment), B like bowel problems and urine incontinences, S like self-care problems (functional dependence), T like tearfulness (mood disorders), A like auditory impairment, C like cognitive impairment, L like low albumin level (malnutrition), E like easy to fall down.

#### Methods of assessment

Binocular visual acuity was tested with Snellen charts for near vision. Vision evaluation was performed as the assessment of functional efficiency of sensory organs with correction of glasses if used.

The examiners used standardized Snellen charts for near vision consisting of eight lines of letters of increasing size (1- the smallest print, 8- the biggest print). If a respondent was unable to read any line of the Snellen scale, he was asked to count fingers shown at a distance instead, and in those severely impaired, the sense of light was checked.

Accordingly, the subjects were classified:

- those with normal vision: respondents who were able to read lines 1-4 at a standard distance,
- those with impaired vision: including those with moderately and significantly impaired vision or even blindness (respondents who were able to read lines 1-4 but failed to do so at the normal distance or read lines 5-8 at any distance, respondents who were able to count fingers and those with preserved sense of light, as well as blind patients).

Vision assessment was not performed among the respondents with end-stage dementia; such respondents were classified as “ineligible” and without a confirmed vision impairment (85 respondents).

Fecal and/or urine incontinences were rated with one of the Activities of Daily Living (ADL) scale questions, namely “bowel and bladder management” [27]. Answers “partially” were classified as “not independent”.

The screening assessment for functional dependence was made with use of the Instrumental Activities of Daily Living Scale (IADL) [28]. Limitation in patient's independence was diagnosed when the respondent scored less than 21 out of 24 points. Answers “partially” were classified as “not independent”.

The screening assessment for cognitive impairment was performed with the Mini-Mental State Examination (MMSE) [29]. Suspicion of cognitive impairment was raised when an examined respondent got 23 points or less out of 30 points. In the case of limitations that might have prevented the respondents from completing certain items of the questionnaire (e.g. motor impairment involving dominant hand, hands tremor, blindness) the percentage of answers possible to receive was calculated. Respondents who were unable to fulfill MMSE due to severe cognitive impairment were classified as end-stage dementia (in that particular cases, descriptions provided by examiners were available; 85 respondents). MMSE was not performed among the respondents with complete deafness; such respondents were classified as “ineligible” and without a confirmed cognitive impairment (three respondents).

Hearing was checked by assessing respondents' ability to hear normal speech and whisper at a distance of three meters. Hearing evaluation was performed as the assessment of functional efficiency of the sensory organs with correction of a hearing aid if used. Respondents were grouped into two categories:

- those with preserved hearing,
- those with any hearing impairment: respondents who were able to hear only loud speech or a single word if spoken very loudly or those not able to hear anything.

Hearing assessment was not performed among those with end-stage dementia; such respondents were classified as “ineligible” and without a confirmed hearing impairment (85 respondents).

Low albumin level was diagnosed when the serum albumin level was below 35g/l (the lower range for normal albumin level used by the laboratory employed in PolSenior Project). The albumin level was measured by colorimetric method (Roche Diagnostics GmbH reagents) and all samples were analyzed in the central laboratory for PolSenior project (SYNEVO Medical Laboratory). For the purpose of this study albumin level below 35g/l was considered, as an index of malnutrition [30].

The screening assessment for mood disorders was performed with Geriatric Depression Scale (GDS, Short Form) [31]. Suspicion of mood disorders was raised if examined respondent got six or more out of 15 points maximum.

GDS was not performed in those with severe cognitive impairment (defined as MMSE below 10 points; 119 respondents) and total deafness (three respondents). These subjects were classified as “ineligible” and regarded as those without a confirmed mood disorders.

On the basis of the questionnaire, history of falling in the last 12 months was collected. A fall was defined as an unexpected incident when participant unintentionally came to rest on the floor or ground or other lower level [32].

### Statistical methods

All analyses were conducted using “svy” commands in STATA 12 software that allowed for adjustment for complex sample designs. Because the study sample was chosen with approximately equal number of respondents in the analyzed gender-age groups, the sample did not reflect strictly the structure of the old (65 and over) population in Poland. In particular, the older age groups and males were overrepresented. To assess the population prevalence of impairments sample weights were applied to produce nationally representative estimates for population aged 65 years and older (according to age and sex structure in Poland in 2009) [33]. All estimates were calculated using those weights to assess the prevalence and co- occurrence of geriatric problems in the population. Prevalence estimates together with 95% confidence intervals were calculated with cross-tabulation. The conditional probability was used to assess the prevalence of one disorder in relation to other disorders.

Logistic regression models were used to estimate prevalence odds ratios (PORs) to assess the strength of relation between two chosen deficits in the whole population as well as in age-groups. Statistical significance was set at p < 0.05 and the stability of the estimates was reflected by 95% confidence intervals (95% CIs)
